# Gestational Exposure to Cyfluthrin through Endoplasmic Reticulum (ER) Stress—Mediated PERK Signaling Pathway Impairs Placental Development

**DOI:** 10.3390/toxics10120733

**Published:** 2022-11-28

**Authors:** Wensi Ni, Haoxuan Gao, Bing Wu, Ji Zhao, Jian Sun, Yanan Song, Yiping Sun, Huifang Yang

**Affiliations:** 1 School of Public Health and Management, Ningxia Medical University, Yinchuan 750001, China; 2 Key Laboratory of Environmental Factors and Chronic Disease Control, Ningxia Medical University, Yinchuan 750001, China

**Keywords:** cyfluthrin, gestational exposure, placental development, endoplasmic reticulum stress, PERK/eIF2α/ATF4/CHOP

## Abstract

Cyfluthrin, a typical type II pyrethroid pesticide, is widely used in house hygiene and agricultural pest control. Several epidemiological investigations have found that maternal pyrethroid exposure is connected to adverse pregnancy outcomes. However, the underlying mechanisms remain to be elucidated. Thus, we evaluated the effect of cyfluthrin exposure during pregnancy on placenta development in vivo. In the current study, Pregnant SD rats were randomly divided into four groups and administered 6.25, 12.5, and 25 mg/kg body weight cyfluthrin or an equivalent volume of corn oil by gavage from GD0 to GD19. The results have shown that gestational exposure to cyfluthrin exerted no effect on the fetal birth defect, survival to PND4, or fetal resorption and death. However, live fetuses and implantation sites significantly decreased in the high-dose cyfluthrin-treated group. Moreover, a significant reduction in placenta weight and diameter was observed in rats. Correspondingly, the fetal weight and crown-rump length from dams exposed to cyfluthrin were reduced. Cyfluthrin-treat groups, the total area of the placenta, spongiotrophoblast area, and labyrinth area had abnormal changes. Meanwhile, the area of blood sinusoid and CD34-positive blood vessel numbers in the placenta were considerably reduced, as well as abnormal expression of placental pro-angiogenic and anti-angiogenic factors in dams exposed to cyfluthrin. Further observation by transmission electron microscopy revealed significant changes in the ultrastructure of the medium-dose and high-dose groups. Additional experiments showed gestational exposure to cyfluthrin inhibited proliferation and induced apoptosis of placentas, as decreased PCNA-positive cells and increased TUNEL-positive cells. Furthermore, western blot and qPCR analysis revealed that gestational exposure to medium-dose and high-dose cyfluthrin increased the expression of GRP78, and three downstream mRNA and proteins (p-eIF2α, ATF4, and CHOP) of the PERK signaling, indicating that endoplasmic reticulum (ER) stress-mediated PERK/eIF2α/ATF4/CHOP signaling pathway in rat placentas was activated. Our study demonstrated that gestational exposure to cyfluthrin leads to placental developmental disorder, which might be associated with ER stress-mediated PERK signaling pathway.

## 1. Introduction

Pyrethroid insecticides are naturally occurring insecticidal compounds derived from the flower Chrysanthemum cinerariifolium [1], which have come to dominate the world market for insecticides as an alternative to organophosphorus pesticides. The reported use of pyrethroid active ingredients fluctuated around a mean value of 7000 tonnes, with two peaks in 1997 (13200 tonnes) and 2012 (16800 tonnes). Geographically, Ukraine, Pakistan, Turkey, Paraguay, and India were the top five countries in the use of pyrethroid active ingredients [2]. Pyrethroid pesticides and their metabolites are detected in urine in 46–79.8% of the general population in America [3]. Recent cohort studies in South Africa [4], French [5], China [6], and Japan [7] have shown detection rates of pyrethroid pesticide metabolites in the urine of pregnant women of 99.7–100%, 100%, 71%–82% and 97.8%, respectively. It also has been detected in breast milk [8], cord blood [9], and as well as in media such as air, water, and sediments environment [10,11]. Depending on whether they contain alpha-cyano, pyrethroid insecticides can be divided into two categories, type I pyrethroids (with low toxicity, used for nonagricultural pest control) and type II pyrethroids (with medium toxicity, used for agriculture pest control). Cyfluthrin, a typical type II pyrethroid, has been widely used in house hygiene and agriculture insect control because of its potent insecticidal efficiency, low mammalian toxicity, and ability to degrade environmentally.

Despite the low toxicity in nontarget organisms attributed to cyfluthrin, the indiscriminate use of it has increased the potentially toxic threats to humans and the environment. Due to the specific physiological effects of pregnant women, who are more sensitive to environmental pollutants and gestational exposure to environmental chemicals can trigger adverse pregnancy outcomes. Therefore, the public health issues caused by cyfluthrin have aroused great concern.

In the past few years, there has been particular concern about pyrethroid exposure in pregnant women, and several epidemiological and toxicological studies have found a negative correlation between gestational pyrethroid exposure and adverse behavior in their children [12,13]. Moreover, a study showed that the risk of children’s developmental delay and autism spectrum disorder are associated with pyrethroid application sites to prenatal maternal residence [14]. Furthermore, low birth weight, social-emotional measures, and lower cognitive abilities were associated with pyrethroid exposure during pregnancy [4]. In addition, the association between these adverse birth outcomes and the subsequent development of mental disorders, diabetes, cardiovascular diseases, and metabolic syndrome is usually referred to as the ‘developmental origin of health and disease, DOHaD’ [15,16]. Some animal studies have suggested that pyrethroid pesticide exposure induces placental damage resulting in intrauterine growth restriction and effects on offspring development, but the possible pathogenesis is unclear [17,18,19].

Abnormal placental function and structure could impair fetal growth and lead to adverse pregnancy outcomes [20]. The placenta is a vital temporary organ, located between the mother and the fetus and has functions like material exchange, defense, synthesis, and immunity. As a highly specialized transient organ, it participates in the dialogue and communication between the fetus and the mother. It plays a crucial role in maintaining fetal growth and development. Gestational exposure to environmental toxicants has been linked to placental injury [21,22]. However, little has been reported on the impacts of cyfluthrin exposure on placental function and development, and the underlying molecular mechanisms of cyfluthrin-induced toxicity in the placenta are not well understood.

The endoplasmic reticulum (ER), one of the most prominent organelles observed in eukaryotes, allows folding maturation and synthesis of secreted and transmembrane cellular proteins under homeostatic conditions. Nevertheless, cellular disturbances triggered by changes in environmental factors or physiological can elicit the ER to experience ER stress. ER stress response is critical to mammalian reproduction by allowing decidualization and placental formation. Prolonged ER stress and activated unfolded protein responses (UPR) pathways can result in cell apoptosis, which could adversely affect pregnancy outcomes and placental formation. PERK/eIF2α/ATF4/CHOP signaling pathway is activated to regulate protein synthesis upon ER stress [23]. PERK can phosphorylate eukaryotic translation initiation factor 2α(eIF2α). The inactivation of eIF2α reduces translational initiation and repression of global protein synthesis [24]. ATF4, a famous transcription factor, regulated multifarious protein synthesis. However, the expression of ATF4 is favored by the eIF2α phosphorylation. When ERs mediate cell apoptosis, CHOP expression in the C/EBP family increases and accumulates in the nucleus, leading to cell cycle arrest and even apoptosis. Previous research has illustrated that ERs disrupt placental development and lead to fetal growth restrictions [25,26]. Few researchers have reported the adverse effect of placental morphogenesis and function after gestational exposure to cyfluthrin. In light of the essential role of the placenta, the investigation of cyfluthrin-induced placental injury needs to be explored. To our knowledge, no detailed investigations are presently available about the effect of cyfluthrin exposure on placenta development during gestation. Hence the purpose of the present study was designed to investigate the effect of gestational exposure to cyfluthrin on placenta development in vivo.

## 2. Materials and Methods

### 2.1. Reagents

Cyfluthrin (purity 98.95%) was bought from Dr. Ehrenstorfer GmbH (CAS NO.68359-37-5, Augsburg, Germany), and the corn oil was purchased from the Shandong Luhua Group Co., Ltd. (Product standard No: Q/LLH 0014S, Yantai, China).

### 2.2. Animals and Experimental Design

The animal use protocol listed below has been reviewed and proved by the Laboratory Animal Ethical and Welfare Committee of the Laboratory Animal Center, Ningxia Medical University (Approval No.: IACUC-NYLAC-2021-101). Both sexes of mature Sprague-Dawley (SD) rats (weighted 240–280 g, aged 10–12 weeks) were bought from Ningxia Medical University Laboratory Animal central (Yinchuan, China). The SD rats live under standard conditions (light/dark alternate for 12 h, 25 °C ± 2 °C temperature, and 50–60% air humidity). Sterilized food and water were accessed ad libitum. In order to induce pregnancy, virgin rats were mated with fertile males at a ratio of 3:1 in the same ventilated polypropylene cage after five days of adaptation. The next morning, the discovery of a vaginal plug was regarded as the gestational day (GD) 0. Pregnant rats were housed individually in ventilated polypropylene cages. Per their body weight, pregnant rats were randomly assigned into four groups (*n* = 10 per group): Vehicle control (corn oil); Low-dose group (6.25 mg/kg); Medium-dose group (12.5 mg/kg); High-dose group (25 mg/kg).

The LD50 of cyfluthrin is reported to be 250 mg/kg body weight in rats [27,28]. For the requirements of experimental design, we chose 6.25 mg/kg (1/40 of the LD50), 12.5 mg/kg (1/20 of the LD50), and 25.0 mg/kg (1/10 of the LD50) as the exposure dose for the present study. Based on the assigned group, the rats were treated with a volume of 0.02 mL/100 g body weight through gavage administration at 9 A.M. continuously from GD1 to GD 19. Maternal body weight was monitored every two days for the study to adjust the perfusion volume and assess pregnancy weight gain. At GD19, a cohort of pregnant rats (*n* = 7 per group) was sacrificed to determine the numbers of litter size, stillbirths, implanted, resorbed, and birth defect embryos. Placental weight, placental diameter, fetal weight, and length were also assessed. Placentae were collected and fixed in glutaraldehyde and 4% paraformaldehyde for histochemical analysis and transmission electron microscopy examination. In addition, placentae were flash-frozen in liquid nitrogen and then stored at −80 °C until further analysis. Natural delivery was allowed for other rats. At postnatal day 0 (PND0), pups were weighed and culled to 12 (six males and six females in each group). The litter size, birth weight, birth defect, and stillbirths number were recorded for each dam, the PND4 survival rate (as an indicator of neonatal health) was calculated, and the pups were sacrificed at six weeks.

### 2.3. Observation of Placental Structure

#### 2.3.1. Hematoxylin-Eosin (HE) Staining

The fresh placentas were fixed in 4% Paraformaldehyde for 24 h (hours). Tissues were used with 50%, 75%, 80%, 90%, and100%ethanol gradient dehydration, transparent with xylene, and embedded in paraffin. Placenta paraffin blocks were cut into 3.5-μm thick sections and stained with hematoxylin and eosin. Tissues were assessed and photographed with a microscope (Olympus, Tokyo, Japan). All measurements were taken using Image J (NIH, Bethesda, MD, USA). The total area of the placenta, the spongiotrophoblast and the labyrinth area, was determined in randomly selected four midline sections of the placenta from the control and treated groups. Four fields were chosen randomly from each section, then quantified the blood sinusoids areas in the labyrinthine region. Calculation of average percentage of blood sinusoid area: the ratio of the area defined by the threshold value to the total number of pixels in the image.

#### 2.3.2. Transmission Electron Microscopy (TEM)

A tissue block of approximately 1 mm^3^ in size was fixed in 2.5% glutaraldehyde for at least 2 h (hours). The tissue block was washed with PBS three times for 15 min each; then, it was fixed in 1% osmium acid for 2 h. Three more washes with PBS for 15 min each. The tissue was dehydrated in gradients of 50%, 70%, 80%, and 90% ethanol for 8 min and 100% ethanol twice for 12 min each. Epoxy resin was embedded, and the tissue blocks were trimmed prior to positioning. After positioning, ultra-thin sections were sectioned and retrieved on a copper grid, stained with uranyl acetate-citrate, observed under the transmission electron microscope (HITACHI, Japan), and photographed.

### 2.4. Enzyme-Linked Immunosorbent Assay (ELISA)

Pregnant rats’ blood was collected and centrifuged (speed: 3500× *g*, duration: 10 min) to obtain maternal serum and then placed at 4 °C. According to the manufacturer’s instruction, the vascular endothelial growth factor A(VEGFa), placental growth factor (PLGF), and FMS-like tyrosine kinase 1(sFLT1) levels in serum were measured using commercially available ELISA kits (J&L biological, Shanghai, China).

### 2.5. Immunohistochemistry (IHC) Staining

The placental sections were deparaffinized in xylene, and dehydrated in gradients of ethanol. Next, the antigen was extracted with ethylene diamine tetra acetic acid antigen extract in a microwave oven.

Then, the sections were incubated in 3% hydrogen peroxide for 18 min to block the activity of endogenous peroxidase, and incubated with the corresponding primary antibodies at 4 °C overnight. Next morning, the sections were incubated with goat anti-rabbit secondary antibody, DAB color reaction, counterstaining with hematoxylin, and assessed and photographed with an optical microscope (Olympus, Tokyo, Japan). Finally, the proportion of PCNA-positive cells and the numbers of CD34-positive blood vessels were analyzed on average in six visual fields in each nonconsecutive section in the placental labyrinth by magnification 40×. The following primary antibodies were used: CD34 (1:800 dilution, Servicebio, Wuhan, China), PCNA (1:500 dilution, Santa Cruz Biotechnology, Santa Cruz, CA, USA).

### 2.6. TUNEL Staining

The TUNEL staining was performed with the TUNEL kit (AF488, Elabscience, Wuhan, China) according to the manufacturer’s instructions. In a nutshell, the placental sections were deparaffinized in xylene, dehydrated in gradients of ethanol, and then permeabilized with Proteinase K. Added TDT equilibration buffer, 37 °C for 30 min and then absorbed the buffer. Put sections into the wet box at 37 °C for 65 min in the dark with a labeled working solution. Washed with PBS and then added DAPI to counterstain the nuclei. Lastly, the sections were sealed with a sealing agent containing anti fluorescence quenching agent and examined with a microscope (Olympus, Tokyo, Japan).

### 2.7. Western Blotting 

The protein lysis mixture was prepared in a 1:100 ratio of phenylmethylsulfonyl fluoride (PMSF) (ST506, Beyotime Biotechnology, Shanghai, China) and immunoprecipitation assay buffer (RIPA) (P0013B, Beyotime Biotechnology, Shanghai, China). Homogenized 50 mg of placental tissue in 500 μL of protein lysis mixture, ground with ultrasound (45 HZ, the 30 s, working 15 s, stopping 2 s), centrifuge (speed: 3500× *g*, time: 5 min) and obtained supernatant, determined the protein concentration with BCA protein concentration assay kit (KGP902, KeyGEN Biotechnology, Nanjing, China). The proteins were separated by SDS-PAGE (PG112, epizyme, Shanghai, China) and then transferred to different groups of PVDF membranes. The membranes were blocked with 3% BSA (Becton, Dickinson and Company, Fulaklin, NY, USA) for 1 h and incubated with VEGFa (ab214424, Abcam, UK), GRP78 (ab21685, Abcam, Cambridge, UK), Phospho-PERK (AbDF7576, Affinity Biosciences, Changzhou, China), PERK (ab2229912, Abcam, Cambridge, UK), Phosphor-eIF2α (3398, CST, Danvers, IL, USA), eIF2α (5324, CST, Danvers, IL, USA), ATF4(11915, CST, Danvers, IL, USA), CHOP (2895, CST, Danvers, IL, USA), β-actin(Servicebio, Wuhan, China) antibody buffer overnight at 4 °C. Dilute the above primary antibody with antibody diluent at a ratio of 1:1000. The following day, the membranes were incubated with goat anti-rabbit and goat anti-mouse secondary antibodies (1:3000 dilution) (Affinity Biosciences, Changzhou, China) at room temperature for 1 h. And then, the membranes were visualized using enhanced chemiluminescence (P0020, Beyotime Biotechnology, Shanghai, China). Image J was used to analyze the relative intensity of the band and with proteins normalized to β-actin.

### 2.8. Total RNA Isolation and Real-Time Quantitative PCR (qPCR)

The total RNA of placenta tissues was extracted and purified using the TRIzol reagent (Invitrogen, Carlsbad, CA, USA). The RNA concentration was measured by Nanodrop one spectrophotometer (Thermo Fisher Scientific, Waltham, MA, USA). The RNA sample was reverse-transcribed into single-stranded cDNA in a 20 μL reaction mixture using the FastKing gDNA Dispelling RT SuperMix (TIANGEN Biotech, Beijing, China). qPCR was performed by GoTaq^®^ qPCR Master Mix (Promega, Madison, WI, USA). A dissociation curve was analyzed after amplification to ensure the purity of PCR products. Sangong Biotech Co. Ltd. (Shanghai, China) designed and synthesized the specific primers for real-time quantitative PCR. These sequences of primers for qPCR were revealed in the Supporting Information, Table S1. Relative gene expression levels were determined using the 2^−ΔΔCT^ method following normalization to β-actin

### 2.9. Statistical Analysis

Statistical analysis was conducted using Prism 8.0 (GraphPad Software, San Diego, CA, USA), and the data were presented as the mean ± SE. Variant values analyses were evaluated by one-way analysis of variance (ANOVA). When significant differences were found between the treatment and control groups, Dunnett’s test was subsequently used for the post hoc multiple comparison test. All the data are based on at least three replications. * *P* < 0.05 and ** *P* < 0.01 were considered as statistically significant.

## 3. Results

### 3.1. Gestational Exposure to Cyfluthrin Disturbs Pregnancy Outcomes and Fetal Development

**Figure 1:**
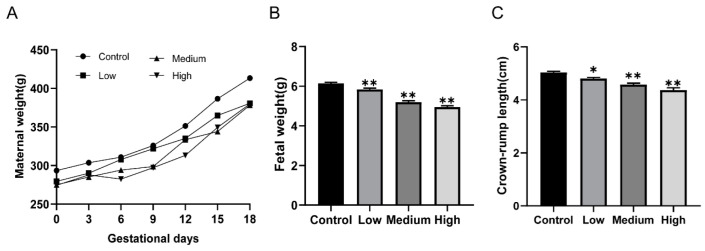
Effects of gestational cyfluthrin exposure on maternal weight, fetal weight, and length. Pregnant rats were administered corn oil or different doses of cyfluthrin (6.25, 12.5, or 25 mg/kg) daily in pregnancy. (**A**) Maternal weight. (**B**) Fetal weight. (**C**) Crown-rump length. Values are expressed as the mean ± SE. The result was analyzed with one-way ANOVA followed by Dunnet’s *t*-test. * *P* < 0.05, ** *P* < 0.01 compared to controls.

To evaluate pregnancy outcomes and fetal development, live fetuses, dead fetuses, implantation sites, the numbers of resorptions, birth defects, and survival to post-natal day (PND)4 per litter were measured. Pregnant rats with relevant doses of cyfluthrin treatment presented markedly reduced gestational body weight (Figure 1A). However, no pregnant rats died after being treated. Next, we analyzed the effect of cyfluthrin-treated on pregnancy outcomes. As described in Table 1, there was no noticeable difference among the groups in the number of dead fetuses, resorption rate, birth defect, and survival to PND4 rate by statistics analysis (Table 1). Although the number of resorbed embryos slightly increased via cyfluthrin treatment, statistical analysis indicated no obvious difference between cyfluthrin-treated groups and the control group (Table 1). However, the number of live fetuses and implantation sites in the high-dose cyfluthrin-treated group markedly decreased compared to the control group (Table 1). Then the effect of gestational cyfluthrin exposure on fetal development was evaluated by measuring fetal weight and crown-rump length. We observed a notable reduction in fetal weight in rats exposed to cyfluthrin during the pregnancy (Figure 1B). Correspondingly, the fetuses’ crown-rump length from dams exposed to cyfluthrin was significantly reduced (Figure 1C). The results showed that maternal exposure to cyfluthrin influenced pregnancy and fetal outcomes.

### 3.2. Gestational Exposure to Cyfluthrin Impairs Placental Development 

**Figure 2:**
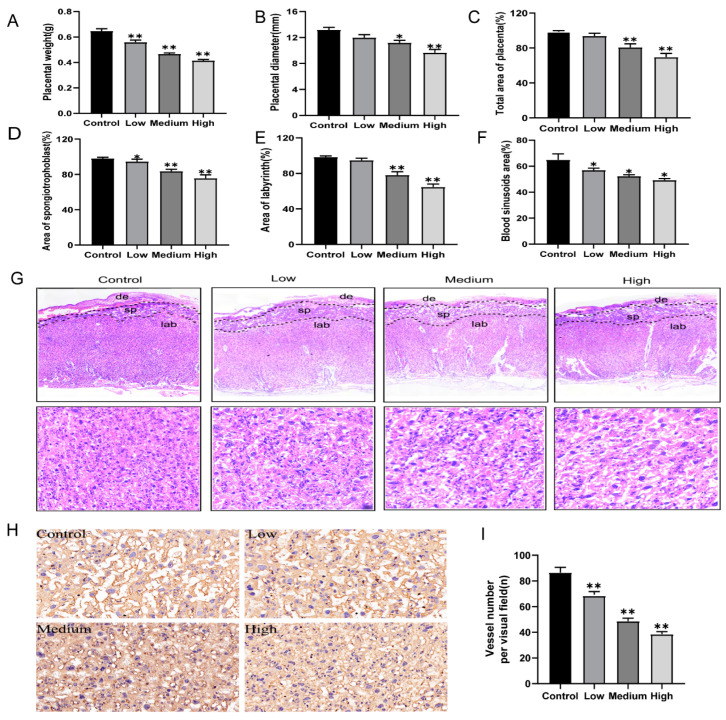
Gestational exposure to cyfluthrin impairs placental development. Pregnant rats were administered corn oil or different doses of cyfluthrin (6.25, 12.5, or 25 mg/kg) daily in pregnancy. Rat placentas were collected on GD19. (**A**) Average placental weight. (**B**) Average placental diameter. (**C**) Total area of placenta(%). (**D**) Area of spongiotrophoblast(%). (**E**) Area of labyrinth(%). (**F**) Blood sinusoid areas in the labyrinthine layer were estimated from five nonconsecutive sections in each placenta. (**G**) Images of placental cross sections, which were stained with H&E. Images of blood sinusoid areas in the placental labyrinthine region. Original magnification: 40×. (**H**) Placental CD34 expression was detected using immunohistochemistry. Representative images were chosen from the placental labyrinth. Original magnification: 40×. (**I**) CD34-positive vessels were counted per visual field. Values are expressed as the mean ± SE. The result was analyzed with one-way ANOVA followed by Dunnet’s *t*-test. * *P* < 0.05, ** *P* < 0.01 compared to controls.

The normal development of the placental is essential for fetal growth. To determine whether cyfluthrin exposure during pregnancy impaired placental development. Placental weight and diameter were measured and analyzed. As illustrated in Figure 2, compared to the control group, placental weight of rats exposed to cyfluthrin during pregnancy markedly decreased dose-dependently (Figure 2A). Furthermore, such reduction of placental diameter was observed too (Figure 2B,C). There are three layers of the rat placenta, including the maternal decidual (de) layer, spongiotrophoblast (sp) layer, and labyrinth (lab) layer. The total area of the placenta was significantly more minor in the high-dose and medium-dose cyfluthrin-treated rats than in the control (Figure 2C). There were observable changes in spongiotrophoblast and labyrinth areas between various doses of cyfluthrin-treated groups and the control group (Figure 2D,E). Moreover, we calculated the blood sinusoid area and the number of blood vessels in the placental labyrinthine region by HE staining and IHC staining for CD34. As illustrated in Figure 2F,G, the blood sinus area in the labyrinthine region of the placenta showed a downward trend in dose-response. Meanwhile, there was a decrease in the number of CD34-positive blood vessels of the placental labyrinth in cyfluthrin-treated rats compared to the control group (Figure 2H,I).

**Figure 3:**
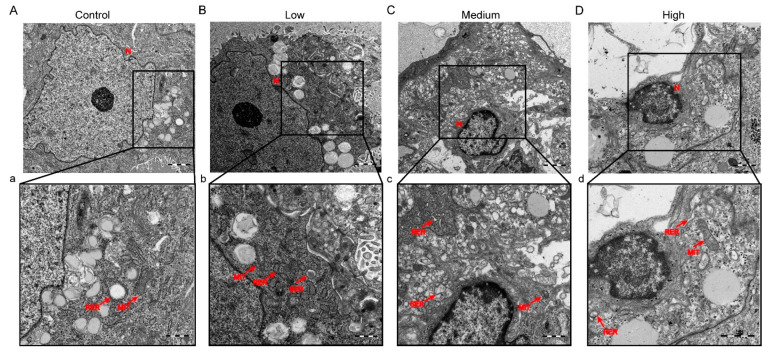
Effects of gestational cyfluthrin exposure on the placental ultrastructure by TEM evaluation. Pregnant rats were administered corn oil or different doses of cyfluthrin (6.25, 12.5, or 25 mg/kg) daily in pregnancy. Rat placentas were collected on GD19. (**A**–**D**) Transmission Electron Microscope images of the placenta in the transverse plane. Original magnification: 20,000×. Scale bars = 2 µm. (**a**–**d**) Transmission Electron Microscope images of the placenta in the transverse plane. Red arrows indicate nucleus, smooth endoplasmic reticulum, rough endoplasmic reticulum, and mitochondrion. Original magnification: 30,000×. Scale bars = 1 µm.

Next, we used a transmission electron microscope (TEM) to examine microscopic changes in the labyrinthine placental region. The cell structure was complete in the control group, and the cytoplasmic organelles were abundant (Figure 3A). Placenta In low-dose cyfluthrin-treated group showed mild dilatation and swelling of the endoplasmic reticulum (Figure 3B). By contrast, analysis of the placenta in the high-dose and medium-dose groups showed significant structural changes by transmission electron microscopic (Figure 3C,D). The nuclear pyknosis, the peripheral heterochromatin became increasingly prominent, mitochondrial swelling, abscission of rough endoplasmic reticulum ribosome, and expansion of sliding endoplasmic reticulum were observed. To sum up, these findings indicated that placenta development was disrupted by cyfluthrin exposure during pregnancy.

### 3.3. Gestational Exposure to Cyfluthrin Alters the Expression Levels of Pro-Angiogenic and Anti-Angiogenic Factors in the Placenta

**Figure 4:**
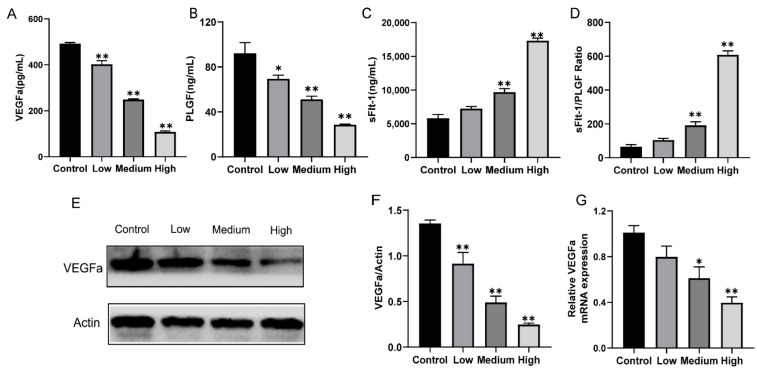
Gestational exposure to cyfluthrin alters levels of the placental anti-angiogenic and anti-angiogenic factors. Pregnant rats were administered corn oil or different doses of cyfluthrin (6.25, 12.5, or 25 mg/kg) daily in pregnancy. Rat serum and placentas were collected on GD19. (**A**) The levels of VEGFa in serum. (**B**) The levels of PLGF in serum. (**C**) The levels of sFlt-1 in serum. (**D**) sFlt-1/PlGF Ratio. (**E**) Western blot image of VEGFa expression. (**F**) Expression levels of VEGFa protein. (**G**) Expression levels of VEGFa mRNA. Values are expressed as the mean ± SE. The result was analyzed with one-way ANOVA followed by Dunnet’s *t*-test. * *P* < 0.05, ** *P* < 0.01 compared to controls.

The formation of the placental vasculature played an essential role in establishing and maintaining pregnancy and fetal development. To determine whether exposure to cyfluthrin altered anti-angiogenic and pro-angiogenic factor secretion levels during pregnancy, serum levels of sFlt-1, VEGFa, and PLGF was measured by ELISA. VEGFa and PlGF, crucial angiogenic factors, showed a significant dose-response decrease compared to the control group expressions (Figure 4A,B). Meantime, there was a markedly decrease in the mRNA and protein expression of VEGFa between cyfluthrin-treated groups and control (Figure 4E–G). Moreover, the expression of sFlt1, an inhibitor of vascular endothelial growth factor-mediated angiogenesis, increased in high-dose and medium-dose groups of rats exposed to cyfluthrin during pregnancy (Figure 4C). The sFlt-1 and PlGF ratio is a biomarker in predicting preeclampsia and poor pregnancy outcomes; the ratio of the high-group significantly increased (Figure 4D). The above results suggest that gestational exposure to cyfluthrin altered the balance between placental pro-angiogenic and anti-angiogenic factors. 

### 3.4. Gestational Exposure to Cyfluthrin Inhibits Cell Proliferation and Induces Cell Apoptosis in the Placenta

**Figure 5:**
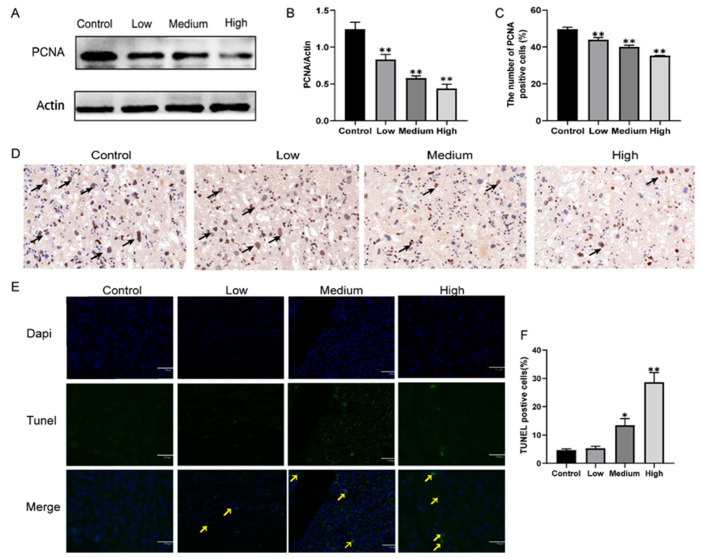
Effects of gestational cyfluthrin exposure on cell proliferation and apoptosis in the placenta. Pregnant rats were administered corn oil or different doses of cyfluthrin (6.25, 12.5, or 25 mg/kg) daily in pregnancy. Rat placentas were collected on GD19. (**A**) Western blot image of PCNA expression. (**B**) Expression levels of PCNA protein. (**C**) Rate of PCNA-positive cells in the labyrinthine region. (**D**) Placenta section was immunohistochemically stained with PCNA. Arrows indicate PCNA-positive cells in the labyrinthine region. Original magnification: 40×. (**E**) TUNEL-labeled placental tissues. Yellow arrows: TUNEL-positive cells. Scale bars = 170 µm. (**F**) The percentage of TUNEL-positive cells. Values are expressed as the mean ± SE. The result was analyzed with one-way ANOVA followed by Dunnet’s *t*-test. * *P* < 0.05, ** *P* < 0.01 compared to controls.

In order to investigate whether the defective placentas were associated with an alteration imbalanced between cell proliferation and apoptosis in the placentas of gestational exposure to cyfluthrin. Cell proliferation was estimated by immunohistochemical staining with PCNA, and cell apoptotic was determined by A TUNEL assay. Our results found that PCNA-positive cells were located in the labyrinthine placental region (Figure 5C). Compared with the control group, cyfluthrin-treated groups markedly reduced the numbers of PCNA-positive cells in the labyrinthine layer in the placentas (Figure 5D). Meanwhile, western blotting illustrated the down-regulation of PCNA protein (Figure 5A,B). Furthermore, there were few TUNEL-positive cells in the rat placentas of the control and low cyfluthrin-treated groups. The percentage of TUNEL-positive cells in the placenta of rats treated with medium-dose and high-dose cyfluthrin was increased. The experiment results indicated that exposure to cyfluthrin during pregnancy inhibits the proliferation of placental cells and induces apoptosis.

### 3.5. Gestational Exposure to Cyfluthrin Triggers ER Stress-Mediated PERK/eIF2α/ATF4/CHOP Signaling in the Placenta

**Figure 6:**
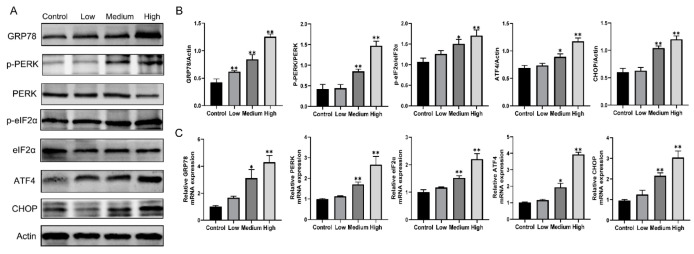
Gestational exposure to cyfluthrin actives ER stress-mediated PERK signaling pathway. Pregnant rats were administered corn oil or different doses of cyfluthrin (6.25, 12.5, or 25 mg/kg) daily in pregnancy. Rat placentas were collected on GD19. (**A**) Western blot image of GRP78, PERK, eIF2α, ATF4, and CHOP expression. (**B**) Expression levels of GRP78, PERK, eIF2α, ATF4, and CHOP protein. (**C**) Expression levels of GRP78, PERK, eIF2α, ATF4, and CHOP mRNA. Values are expressed as the mean ± SE. The result was analyzed with one-way ANOVA followed by Dunnet’s *t*-test. * *P* < 0.05, ** *P* < 0.01 compared to controls.

To further determine whether different doses of cyfluthrin exposure triggered ER stress, the critical proteins GRP78 of ER stress in the placenta tissue were measured. The mRNA and protein expression of GRP78 significantly increased compared to the control, and dose-response relationships were observed (Figure 6). Then, we used qPCR and western blot analysis to calculate if the PERK signaling pathway was associated with cyfluthrin exposure. Figure 6 shows that the expression of phosphorylated PERK (p-PERK), phosphorylated eIF2α (p-eIF2α), ATF4, and CHOP have significantly increased in gestational exposure to medium-dose and high-dose groups of cyfluthrin compared with the control expression. In summary, the results of our study demonstrated that gestational exposure to cyfluthrin activated ER stress-mediated PERK signal pathways.

## 4. Discussion

The epidemiological investigation reported that maternal exposure to pyrethroid during gestation is linked to adverse pregnancy outcomes and poor offspring development [4,6,12,13]. Fetal development is a complex dynamic process. The placenta is a crucial temporary organ linking the mother to the fetus, which plays an essential role in maintaining pregnancy and fetal development. However, it is rarely investigated that the effect of exposure to cyfluthrin during pregnancy on placental development and the biological mechanisms are poorly understood. So far as we know, this study is the first comprehensive investigation to address the effects of gestational exposure to different doses of cyfluthrin on placenta development.

No statistical difference was analyzed in the number of dead fetuses, resorption rate, birth defect, and survival to PND4 rate of gestational exposure cyfluthrin. Although there was a small increase in the number of resorbed embryos of cyfluthrin-treat groups, statistical analysis indicated no apparent difference between cyfluthrin-treated groups and the control group. However, compared with the control group, the number of live fetuses and implantation sites markedly decreased in the high-dose cyfluthrin-treat group. In addition, the result of our study verified that gestational exposure to relevant doses of cyfluthrin lowered the fetal weight and crown-rump length in rats.

Adverse pregnancy outcomes could be attributed to abnormal placental development. The placenta is a pregnancy-specific structure that occurs simultaneously with the development of the embryo and fetus. As a unique organ of mammals during pregnancy, the placenta has various functions to support fetal development, including nutrient transport, waste elimination, sufficient blood supply, synthesis and secretion of pregnancy-related hormones, and barrier function [29].

We found significantly reduced placenta weight and diameter resulting from gestational cyfluthrin exposure. Correspondingly, fetal body weight and crown-rump length from dams exposed to cyfluthrin were significantly reduced. Therefore, it is reasonable to assume that lower fetal weight and crown-rump length might attribute to placental dysplasia in gestational cyfluthrin exposure. Some studies have found that exposure to environmental toxins during pregnancy could induce placental dysfunction, leading to poor pregnancy outcomes, including fetal growth restriction [22,30]. The efficiency of the placenta has been shown to depend to some extent on morphological alterations to the placenta. The junctional area is composed of spongiotrophoblasts, trophoblasts, and trophoblast giant cells (TGCs), which separate the maternal decidua from the labyrinth region [31]. The decidua structure can regulate the spiral artery remodeling and invasion of trophoblast cells. The labyrinth zone delivers nutrients and gas to the developing fetus, while the spongiotrophoblast area has energy storage and endocrine functions [32]. The reduction of the placental surface area is an important factor leading to placental dysfunction and hence FGR [33]. Consistent with other studies, the placental insufficiency models could identify changes in the relative size of placental regions [34,35]. Our result showed that exposure to relevant cyfluthrin during the gestation alters the placenta structure, which reduced the total area of the placenta, the area of the spongiotrophoblasts, and the area of the labyrinth, compared to the control.

Placental labyrinth comprises numerous blood vessels and is the place of oxygen and nutrient exchange between maternal and fetal [36]. The loss of the labyrinth sinusoid area of the placenta directly impacts the materials transport of the mother and fetus. Some studies of environmental pollutant exposure leading to fetal growth restriction have shown a reduction in the blood sinusoid area in the placental labyrinth [17,26]. Our study found that the blood sinusoid area of the placental labyrinth in rats exposed to different doses of cyfluthrin was reduced by HE staining. Since the blood sinusoid area of the placental labyrinth was the zone where nutrient and gas exchange between fetal and maternal blood, the reduction of the blood sinusoid area indicates that the transport of nutrients in the placenta was inhibited. In addition, the present study also used CD34 to assess microvascular density in the labyrinthine region. CD34 is a potential indicator of vascular differentiation expressed by embryonic hematopoietic and endothelial cells [37]. As determined by CD34 immunostaining, a noticeable reduction in placental microvessels was detected in rats exposed to cyfluthrin during gestation. The transmission electron microscope analysis found that nuclear pyknosis, noticeable dilatation of mitochondria, abscission of rough endoplasmic reticulum ribosomes, and expansion of sliding endoplasmic reticulum were observed in the medium-dose and high-dose placental tissues.

Successful pregnancies and normal placenta development were linked to proper vascularization in the placental labyrinth [38]. Vascular endothelial growth factor family plays an essential role in the regulation of angiogenesis, and VEGFa is represented by seven of its encoded members. PlGF is secreted by trophoblasts and is another member of the VEGF family that participates in vasculogenesis and vasodilation by binding to sFlt-1 [39]. The sFlt-1 is an inhibitor of vascular endothelial growth factor-mediated angiogenesis. Specifically, sFlt-1 is thought to interfere with placental vascular development by combing VEGFa and PLGF to inhibit their biological functions [40]. High expression levels of sFlt-1 may lead to serious consequences, like fetal growth restriction through vasoconstriction and endothelial damage [41]. In our study, the expressions of VEGFa and PlGF showed a significant dose-response decrease compared to the expression of the control group. Meanwhile, the mRNA and protein expression of VEGFa markedly decreased compared to the control. The endothelial dysfunction and the decreased cell proliferation may cause by the deregulation of PlGF and VEGFa, generating a placental dysfunction [42]. In addition, gestational cyfluthrin exposure increased both high-dose and medium-dose expression of sFlt1. As a biomarker for predicting adverse pregnancy outcomes and preeclampsia, the sFlt-1 and PlGF ratio was higher in the high-dose cyfluthrin-treated group. These results suggested that gestational exposure to cyfluthrin may break the balance between angiogenic and antiangiogenic factor expression, seriously damaging vascular function during the development of the placenta.

We further observed whether gestational cyfluthrin exposure to developmental disorders of the placenta was linked to cell proliferation and apoptosis alters of the placenta. The development of the placenta requires a high level of cell proliferation to form new tissue and a controlled organization of the tissue to perform the oxygenation and nourishment of the fetus properly. In the present study, gestational exposure to cyfluthrin significantly inhibited placental proliferation by downregulation of PCNA protein. Meanwhile, Immunohistochemistry illustrated the downregulation of PCNA-positive cells. Cell apoptosis, a programmed form of cell death, plays a vital role in placental growth and development. In the early stage of placental development, increased apoptosis helps the placenta invade the endometrium and establishes perfect maternal-fetal circulation. Later in placental development, apoptosis is significantly reduced to avoid excessive invasion of the placenta [43]. We found that gestational high-dose cyfluthrin markedly increased the number of TUNEL-positive cells in the rat placenta. These findings suggested that gestational exposure to different doses of cyfluthrin inhibits the proliferation and induces apoptosis in the placental and may be responsible for the impaired placental structure and growth disorder.

The mechanism by which exposure to cyfluthrin during pregnancy impairs placental development remains unclear. Previous research on animals and humans has found that ER stress is essential for placental development and mammalian reproduction [25,44]. The endoplasmic reticulum (ER), one of the largest organelles observed in eukaryotes, has the primary function of protein synthesis, modification, and processing under homeostatic conditions. When a large amount of unfolded proteins accumulate in the ER lumen, ER stress will be triggered, leading to ER stress and, consequently, the unfolded protein response (UPR) [42]. However, the physiological regulation of UPR directly affects placental development. Recently, several studies reported that long-term ER stress-induced placental dysplasia slows the proliferation, downregulating the expression of the placental growth factors and causing reduced placental villous volume [45,46]. Moreover, when prolonged endoplasmic reticulum stress leads to uncorrected ER dysfunction, cells can initiate apoptosis, eventually leading to cell death [47].

To explore whether the cyfluthrin-induced placental injury is linked to ER stress, we measured the expression of protein and mRNA expression levels of GRP78, a molecular chaperone in the ER cavity, and represents a marker of ER stress activation. As predicted, the GRP78 protein and mRNA expression levels were noticeably upregulated compared to the control group in the rat placenta. It suggested that gestational cyfluthrin exposure could trigger ER stress in the rat placenta. Upon ER stress, the UPR signal transducers and subsequent downstream signals were activated. The type I transmembrane protein PERK played a central role in ER stress. When PERK is separated from GRP78, activating its oligomerization and autophosphorylation structural domains in the cytosolic kinase and phosphorylating the translation initiation factor eIF2α to inactivate. The inactivation of eIF2α results in a reduction of translational initiation and repression of global protein synthesis. The expression of the famous transcription factor ATF4 is favored by the eIF2α phosphorylation. However, ATF4 translocates to the nucleus and induces gene transcription required to alleviate ER stress. CHOP is an essential mediator in evaluating ER-mediated apoptosis by regulating the expression of numerous proapoptotic and antiapoptotic genes [48]. CHOP was predominantly up-regulation through the phosphorylation of eIF2α and the up-regulation of ATF4 expression [49]. We found that exposure to high-dose and medium-dose cyfluthrin significantly upregulated the protein levels of phosphorylated PERK (p-PERK), phosphorylated eIF2α (p-eIF2α), ATF4, and CHOP expression compared to the control expression. The changes in ER stress-related mRNA levels further supported the changes in protein levels. Our study found that exposure to different doses of cyfluthrin during gestation could induce placenta injury by ER stress-mediated PERK/eIF2α/ATF4/CHOP signaling pathway.

## 5. Conclusions

In conclusion, the present study investigated the effect of gestational exposure to relevant doses of cyfluthrin on the placenta and fetal development. Our findings provided robust evidence that cyfluthrin exposure during pregnancy can result in abnormal placental development in rats, which have “ripple effects” that result in adverse pregnancy outcomes, including impairing placental development, dysregulating placental vascularization and angiogenesis, inhibiting the proliferation of placenta, and inducing apoptosis of placenta in the rat. Additionally, we found that the ER stress-mediated PERK/eIF2α/ATF4/CHOP pathway was strongly associated with placental injury induced by cyfluthrin. It was suggested that endothelial dysfunction, decrease in cell proliferation, and increase in cell apoptosis derived from ER stress are the major triggering factors for placenta development. These data provide novel perspectives for revealing the etiology of cyfluthrin-induced reproductive and developmental toxicity and have profound implications for public health prevention. Moreover, the mechanism of cyfluthrin-induced placenta dysfunction needs to be elucidated in further work.

## Figures and Tables

**Table 1:** Effects of gestational cyfluthrin exposure on fetal outcomes and fetal development (mean ± SE.).

	Indexes	Control	Low	Medium	High
Groups					
Birth defect per litter (n)		0	0	0.29 ± 0.76	0.14 ± 0.38
Dead fetuses per litter (n)		0	0.14 ± 0.38	0.29 ± 0.49	0.43 ± 0.79
Resorptions per litter (n)		0.14 ± 0.14	0.29 ± 0.29	0.57 ± 0.29	0.86 ± 0.40
Implantation sites per litter (n)		12.14 ± 0.43	12.00 ± 0.38	11.57 ± 0.53	10.57 ± 0.69 *
Live fetuses per litter (n)		12.86 ± 0.51	12.57 ± 0.37	11.57 ± 0.48	10.43 ± 0.72 *
Survival to PND4 (%)		100	100	95.97	98.5

* *P* < 0.05, compared to controls.

## Supplementary Materials

The following supporting information can be downloaded at: https://www.mdpi.com/article/10.3390/toxics10120733/s1, Table S1: Primer sequences of genes.

## Abbreviations

The following abbreviations are used in this manuscript:

de	decidua
sp	spongiotrophoblast
lab	labyrinth
TEM	transmission electron microscope
N	nuclear
MIT	mitochondrion
SER	smooth endoplasmic reticulum
RER	rough endoplasmic reticulum
