# Supplementary material for: Gestational Exposure to Cyfluthrin through Endoplasmic Reticulum (ER) Stress—Mediated PERK Signaling Pathway Impairs Placental Development
Source: Toxics. 2022 Nov 28;10(12):733. doi: 10.3390/toxics10120733 (PMC9783295; doi:10.3390/toxics10120733)
Supplement: Supplementary file 1 [file toxics-10-00733-s001.zip › toxics-2012369-supplementary.pdf]

toxics

MDPI

Gestational Exposure to Cyfluthrin through Endoplasmic Reticulum (ER) Stress—Mediated PERK Signaling Pathway Impairs Placental Development

Wensi Ni [1,2], Haoxuan Gao [1,2], Bing Wu [1,2], Ji Zhao [1,2], Jian Sun [1,2], Yanan Song [1,2], Yiping Sun [1,2] and Huifang Yang [1,2,*]

Table S1. Primer sequences of genes.

genes		Primer sequence
**GRP78**	Forword	CCAACGAGTAGCGAGTTCACCAATC
	Reverse	GCCAAGTGTCCAAGTCAGTGTAGTC
PERK(Eif2ak3)	Forword	TGGGATGTCGCCGATGGGATAG
	Reverse	AATTCCACTTCTCACTGCCGCTTC
eIF2α	Forword	GCGAATTGTGGCAGGTTTCTTGG
	Reverse	TAGGCTCCTCACTAGGCACTTCAC
ATF4	Forword	CTGCTTGCTCTGTGGTAGATGTCTC
	Reverse	CTCTGCTGCCTCTAATACGCCATG
CHOP	Forword	TGGCATCACCTCCTGTCTGTCTC
	Reverse	CCCTCTCCTTTGGTCTACCCTCAG
VEGFa	Forword	GCACTGGACCCTGGCTTTACTG
	Reverse	ATCGGGGTACTCCTGGAAGATGTC
